# Evaluating clonidine and buprenorphine as adjuvants to intrathecal 0.5% hyperbaric bupivacaine in lower limb surgeries

**DOI:** 10.6026/973206300212595

**Published:** 2025-08-31

**Authors:** Sushma Singh, Sushma Naidu V., Gautam Chandra Koshi

**Affiliations:** 1 Department of Anaesthesia, Government Medical College, Korba, Chhattisgarh, India; 2 Department of Pharmacology, PES University Institute of Medical Sciences and Research, Bangalore, Karnataka, India; 3 Department of Anaesthesiology, Gandhi Medical College, Bhopal, Madhya Pradesh, India

**Keywords:** Clonidine, buprenorphine, intrathecal, bupivacaine, lower limb surgery

## Abstract

Intrathecal clonidine and buprenorphine as adjuvants to 0.5 percent hyperbaric bupivacaine in lower limb surgery is of interest. Patients were randomly allocated to three control groups, each comprising 30 individuals. The analgesic duration was greatest in Group Bu (buprenorphine 280 +/- 20 min) compared to Group C (clonidine 250 +/- 25 min) and Group B (control 180 +/- 22 min). The clonidine group had increased sedation. Buprenorphine showed reduced damage and minor adverse effects.

## Background:

Spinal anaesthesia remains one of the commonly used anaesthetic methods, especially in surgery of the lower limbs, because it is easy to predict, simple and provides excellent sensory and motor block. It provides superb intraoperative addictive conditions with minimal systemic effects and it also comes with a bonus of the prevention of perioperative complications in the form of blood loss and thromboembolic events [1]. Of all the local anaesthetics applied intrathecally, 0.5% hyperbaric bupivacaine is the current agent of choice due to its desirable pharmacokinetics profile, long duration of action, and limited occurrence of neurotoxicity [1]. Nevertheless, bupivacaine is by itself frequently inadequate to produce prolonged post-anaesthesia discomfort, particularly of greater or more painful operations. This has seen the rise in the use of intrathecal adjuvants that have the power to augment both the quality and duration of anaesthesia [2]. The interaction of local anaesthetics with adjuvants like opioids and 2-adrenergic agonists has already been examined considerably, as they are synergistic. A very potent and lipophilic partial agonist of the opioid receptor (mu), buprenorphine, exhibits high affinity to the receptor and long activity time, thus leading to the prolonged analgesic effect after the intrathecal administration [3]. Due to low dissociation rate and a negligible degree of euphoria, buprenorphine is found to be an effective pain reliever post-surgery with comparatively fewer cases of respiratory depression [4]. According to clinical testing, intrathecal buprenorphine has been found to considerably increase the duration of sensory block and use less rescue analgesics [5].

Conversely, Clonidine is a selective Alpha 2 adrenoreceptor agonist that improves spinal anaesthesia, which is achieved by preventing the release of nociceptive neurotransmitters in the dorsal root of the substantia gelatinosa. It also helps in neuronal hyperpolarization, thus enhancing the effects of local anaesthetics [6]. When administered as an intrathecal drug, clonidine has been demonstrated to prolong the sensory and motor blockade as well as enhance the quality of overall postoperative analgesia [7]. Nevertheless, it does not come without a negative side. It has sympatholytic activity that may result in its low blood pressure, bradycardia and sedation, especially at a higher dose [8]. Even though several studies have evaluated the separate usefulness of clonidine and buprenorphine as adjuvants during the use of spinal anaesthesia, there is not a large body of data comparing the relative effectiveness and side effect profile of both agents in a controlled clinical setting [9]. The differential effect of these agents is vital in how to choose the most suitable adjuvant depending on the needs of various surgeries and different risk aspects of the patient. This study was conducted to assess and compare the effectiveness of clonidine and buprenorphine as adjuncts to 0.5% hyperbaric bupivacaine in spinal anaesthesia for lower limb procedures. The principal outcomes evaluated encompass the initiation and duration of sensory and motor blockage, the length of effective postoperative analgesia, and the occurrence of side effects including hypotension, bradycardia, nausea, vomiting, and drowsiness [9]. Therefore, it is of interest to evaluate clonidine and buprenorphine as adjuvants to intrathecal 0.5% hyperbaric bupivacaine in lower limb surgeries.

## Materials and Methods:

This prospective randomised, double-blind, comparative investigation was done with institutional ethical committee permission and written informed consent from all participants. Ninety adult patients (ASA physical status I or II), aged 18 to 60 years, were enrolled for elective lower limb orthopaedic procedures under spinal anaesthesia.

Patients were randomly divided into three equal groups (n = 30) using a computer-generated randomization table:

[1] Group B (Control): Received 3 ml of 0.5% hyperbaric bupivacaine (15 mg) + 0.5 ml normal saline intrathecally

[2] Group C (Clonidine): Received 3 ml of 0.5% hyperbaric bupivacaine (15 mg) + 30 µg clonidine (diluted to 0.5 ml with saline)

[3] Group Bu (Buprenorphine): Received 3 ml of 0.5% hyperbaric bupivacaine (15 mg) + 60 µg buprenorphine (diluted to 0.5 ml with saline)

The volume administered intrathecally in each group was 3.5 ml. A standard monitoring protocol, including non-invasive blood pressure measurement, electrocardiography, and pulse oximetry, was established. The baseline haemodynamic parameters were recorded. Anaesthesia was administered by a spinal procedure performed in a seated position at the L3-L4 interspace using a 25G Quincke needle. Upon confirmation of unobstructed cerebrospinal fluid flow, the study medication was administered over duration of 10 to 15 seconds. The sensory block was assessed using the pinprick method, whereas the motor block was examined with the modified Bromage scale. Documentation was conducted about the onset of sensory and motor blockade, the duration of the blockade, the duration of effective analgesia (the interval between spinal block administration and the initial analgesic intervention), and haemodynamic fluctuations. Nausea, vomiting, and drowsiness (assessed by the Ramsay drowsiness Scale), together with hypotension (SBP <90 mmHg) and bradycardia (HR <50 bpm), were documented. Intraoperative treatment for all patients involved identical administration of fluids and oxygenation. Postoperative analgesia was administered via intravenous paracetamol 1 g as a rescue medicine. The data analysis was conducted using SPSS software version 25. All quantitative results were presented as mean ± SD and analysed using a one-way analysis of variance followed by a post hoc Tukey test. The Chi-square test or Fisher's exact test was employed to examine categorical variables. The chosen statistically significant threshold was a p-value of less than 0.05.

## Results:

Ninety patients were included and evenly distributed into three groups (n = 30 each group). The demographic data, including age, gender, weight, and operation duration, were consistent across all groups, exhibiting no statistically significant difference (p > 0.05) (Table 1 - see PDF). The initiation of sensory block occurred most rapidly in Group C (3.8 ± 0.7 min), succeeded by Group Bu (4.3 ± 0.6 min) and Group B (4.6 ± 0.5 min), exhibiting a statistically significant difference among the groups (p < 0.01). The initiation of motor block occurred earlier in Group C (5.5 ± 0.8 min) than in Group Bu (6.2 ± 0.7 min) and Group B (6.5 ± 0.9 min) (Table 2 - see PDF). The sensory block duration was largest in Group Bu (282 ± 18 min), followed by Group C (256 ± 22 min), and smallest in Group B (184 ± 20 min). The duration of effective postoperative analgesia was markedly greater in Group Bu (410 ± 25 min) than in Group C (370 ± 20 min) and Group B (220 ± 18 min) (Table 3 - see PDF). Sedation was more significant in Group C, with 70% of patients exhibiting a Ramsay Sedation Score ≥3, as contrast to 30% in Group Bu and 10% in Group B. The occurrence of hypotension and bradycardia was elevated in Group C, at 20% and 13.3%, respectively, in comparison to the other groups. Group Bu exhibited a reduced incidence of adverse events overall (Table 4 - see PDF). These results (Tables 1-4 - see PDF) suggest that while clonidine leads to a faster onset of block and more sedation, buprenorphine provides a significantly longer duration of postoperative analgesia with fewer adverse effects.

## Discussion:

This study has compared the action of the two intrathecal adjuvants, clonidine and buprenorphine, in lower limb surgery patients in the treatment with 0.5 percent hyperbaric bupivacaine. Its results indicate that clonidine and buprenorphine have improved and prolonged the quality and duration of the spinal anaesthesia when compared to the same, but with bupivacaine alone, significantly improved the postoperative analgesia, and have a better side-effect profile with buprenorphine. The onset of sensory and motor block was shown to be much more rapid in the clonidine group. This aligns with prior data indicating that clonidine facilitates a more rapid start of neuraxial blockade through its effects on presynaptic and postsynaptic alpha-2 adrenergic receptors in the spinal cord's dorsal horn [1, 2]. These receptors enhance pain transmission by inhibiting the release of nociceptive neurotransmitters and promoting neuronal hyperpolarisation, hence augmenting the efficacy of local anaesthetics [3, 4]. The partial agonist of the opioid mu receptor, characterized by a high degree of lipid solubility and an extended residence in the spinal cord, *i.e.*, buprenorphine, produced an exceptionally longer effect on sensory block and postoperative analgesia in our study. These findings prove to be consistent with the previous results in which the use of intrathecal buprenorphine provided significant analgesic duration without raising the chance of respiratory depression or prolongation of motor block [5]. That is why it is the choice of agent during surgical operations, where good pain control during a long period after the surgery is needed. Whereas the sensory and motor block duration was also increased with the use of clonidine in comparison with the use of bupivacaine alone, it was less significant than with buprenorphine. The adverse effects of clonidine hypotension, bradycardia, sedation, and others, were more common and can be due to the absorption of clonidine into the systemic circulation and its central sympatholytic effect [7, 8]. Findings on these outcomes are complemented by other research findings on hemodynamic disturbances secondary to the administration of intrathecal clonidine that are dose-dependent [9, 10]. The duration of analgesia in the case of buprenorphine corroborates its use as a good and dependable intrathecal adjuvant. It has a great affinity to receptors and a slow dissociation rate that guarantees long-lasting analgesia and eliminates the necessity of a rescue analgesic. In addition, there is a low chance of tolerance and dependence since there is a partial agonist effect [11, 12]. These findings are in agreement with Joseph *et al.* who, reported that buprenorphine provided the longest duration of postoperative analgesia with fewer adverse effects compared to clonidine [13]. It has been previously observed that buprenorphine can readily extend analgesia in low doses with no significant rise in side effects (*e.g.*, pruritus or respiratory depression) [14, 15]. Our results also indicate that clonidine has a greater intraoperative sedation effect as compared to buprenorphine. Although this can be favourable during surgery due to decreased anxiety and motor activity, the effect can be undesirable in ambulatory settings or postoperative rehabilitation, especially in geriatric or unstable hemodynamic patients [16, 17]. The increased Ramsay scores of sedation in the clonidine group indicate that 12-agonists exert sedative actions through locus coeruleus in the brainstem [18, 19]. The nausea and vomiting rate was not particularly high and consistent in all groups and more to the point, none of the patients in any group experienced respiratory depression. Such findings agree with the previous reports, which have assessed the safety of low-dose intrathecal clonidine and buprenorphine [20, 21].

## Conclusion:

The use of intrathecal adjuvants in enhancing spinal anaesthesia is shown. Although clonidine and buprenorphine are effective, buprenorphine has a greater analgesic duration with fewer side effects. Thus a more preferable drug for surgeries is required to manage postoperative pain in the long term.
